# Factors influencing nurses' compliance with Standard Precautions in order to avoid occupational exposure to microorganisms: A focus group study

**DOI:** 10.1186/1472-6955-10-1

**Published:** 2011-01-21

**Authors:** Georgios Efstathiou, Evridiki Papastavrou, Vasilios Raftopoulos, Anastasios Merkouris

**Affiliations:** 1 Department of Nursing, School of Health Science Cyprus University of Technology, Limassol, Cyprus; 2 Department of Nursing, School of Health Sciences Cyprus University of Technology, Limassol, Cyprus; 3 Department of Nursing, School of Health Sciences Mediterranean Research Centre for Public Health and Quality of Care Cyprus University of Technology, Limassol, Cyprus; 4 Department of Nursing, School of Health Sciences Cyprus University of Technology, Limassol, Cyprus

## Abstract

**Background:**

Nurses may acquire an infection during the provision of nursing care because of occupational exposure to microorganisms. Relevant literature reports that, compliance with Standard Precautions (a set of guidelines that can protect health care professionals from being exposed to microorganisms) is low among nurses. Additionally, high rates of exposure to microorganisms among nurses via several modes (needlesticks, hand contamination with blood, exposure to air-transmitted microorganisms) occur. The aim of the study was to study the factors that influence nurses' compliance with Standard Precaution in order to avoid occupational exposure to pathogens, by employing a qualitative research design.

**Method:**

A focus group approach was used to explore the issue under study. Four focus groups (N = 30) were organised to elicit nurses' perception of the factors that influence their compliance with Standard Precautions. The Health Belief Model (HBM) was used as the theoretical framework and the data were analysed according to predetermined criteria.

**Results:**

Following content analysis, factors that influence nurses' compliance emerged. Most factors could be applied to one of the main domains of the HBM: benefits, barriers, severity, susceptibility, cues to action, and self-efficacy.

**Conclusions:**

Changing current behavior requires knowledge of the factors that may influence nurses' compliance with Standard Precautions. This knowledge will facilitate in the implementation of programs and preventive actions that contribute in avoiding of occupational exposure.

## Background

Health care professionals and particularly nurses are often exposed to microorganisms, many of which can cause serious or even lethal infections [1-3]. In 1996, the Centers for Disease Control and Prevention (CDC) issued the Standard Precautions, a set of guidelines to prevent exposure [4], but unfortunately, despite the simplicity and clarity of these guidelines, compliance among nurses is reported low [5-8]. Although high incidence of occupational exposure to microorganisms is observed among all health care professionals [9-11], nurses are among those who are more highly exposed [12]. Therefore it is ethical to explore the factors that affect nurses' compliance with Standard Precautions.

### Prevention of occupational exposure

Occupational exposure can occur in different modes. These modes include contact (direct and indirect) transmission, droplet transmission, airborne transmission, percutaneous exposure and mucus membranes exposure. Many pathogens may share more than one [4,13].

In 1970, the first set of preventive guidelines was issued by the CDC to help health care professionals protect themselves and patients from the transmission of microorganisms, followed by a revision in 1983. In 1987, the Universal Precautions were released. They, among others, required health care professionals to treat every patient as potentially infectious [2,14]. These guidelines were again revised with more details as to when they should be applied [14]. In 1990, the Body Substance Isolation practice was described [15], which required the use of protective equipment (similar to those described in Universal Precautions) in all cases when exposure was anticipated. In 1996, the CDC, in order to clarify the different instructions, which in some cases seemed to cause confusion, issued the Standard Precautions by combining the main principles of Universal Precautions and Body Substance Isolation practice [4].

### Requirements for implementation of Standard Precautions in Cyprus

Health care workers in Cyprus are obliged by law (law 89(I)/96) [16] to implement all components of Standard Precautions during their clinical practice, and take all the appropriate measures to avoid occupational exposure to pathogens. In addition, employers in Cyprus (including hospital managers) are also obliged by the same law to provide to their employees all the necessary means (e.g. gloves, face masks) for protecting their health. Special educated personnel (doctors and nurses) in hospitals, representing the central infection control committee of the Ministry of Health within each hospital, is responsible for training health care workers, and monitor the implementation of Standard Precautions.

### Compliance

Compliance has been defined in many ways [17,18]. Heynes et al [19] offered a widely accepted definition of compliance within health care settings [20]. According to this definition, compliance is the extent to which certain behaviour (for example, following physician's orders or implementing healthier lifestyles) is in accordance with the physicians' instructions or health care advice. Compliance can be influenced or controlled by a variety of factors like culture, economic and social factors, self-efficacy, and lack of knowledge or means. Guidelines that guide an individual's behaviour exist in a variety of settings (including health care settings), but people do not always comply with them. In order to explain and understand the factors that influence an individual's compliance with certain guidelines, which consequently may contribute to the adoption of certain behaviour, a number of conceptual models or theories have been developed. One of the most commonly used models is the HBM [21,22].

### Compliance with precautions among nurses

Studies have shown that compliance with precautions among nurses in order to avoid exposure to microorganisms is low. More specifically, compliance was found inadequate concerning hand hygiene guidelines [23,24], use of gloves when exposure to body fluids was anticipated [10,23-25], eye protection [6,7,26,27], mouth and nose protection (mask use) [6,7,10,26], wearing a gown when required [7,10,27], avoid recapping the needle after it was used for a patient [10], and provision of care considering all patients as potentially infectious [28,29].

### Factors leading to non-compliance

Many researchers focused on the factors that contribute to non-compliance with Standard Precautions. Reported factors were lack of knowledge [30,31], lack of time [7,25,29,30], forgetfulness [30,31], lack of means [30,31], negative influence of the equipment on nursing skills [8,23,25,29], uncomfortable equipment [25,29], skin irritation [31], lack of training [27], conflict between the need to provide care and self-protection [27] and distance to necessary equipment or facility [31]. Compliance with precautions has been studied by using a variety of methods, including questionnaire distribution. In many cases, there was no theoretical framework behind these questionnaires and mostly factors that led to noncompliance were studied. Only few studies incorporated a theoretical framework, however, most of them studied only one or limited aspects of Standard Precautions, mainly hand hygiene [8,32-35].

### Health Belief Model

HBM has been widely used and is considered as one of the most useful models in health care prevention and promotion [36]. It offers the ability to understand the different behaviours or attitudes people may develop under the same condition by following or not following certain guidelines or requirements [37]. The model was originally developed by four psychologists, Hochbaum, Kegels, Rosenstock and Leventhal in the 1950s as a way to examine the reasons that prevented people from using free programs, which would detect or prevent diseases [38]. The original model had four constructs, supplemented later by more (table 1) [39-41]. It is based on two axes: a) the perceived threat for acquiring a disease, which incorporates the perceived susceptibility and perceived severity constructs. This axis creates a pressure to an individual for action, nevertheless this action may not necessarilly take place [42] and b) the enabling factors that trigger the behaviour, which include the perceived benefits and perceived barriers. The additional constructs were supplemented later in order to overcome some limitations the model showed. Therefore, the self-efficacy and cues to action were added [43]. In addition, when using this model, other factor like social and demographics factors must be taken into account.

**Table 1:** Constructs of HBM

• Susceptibility: personal perception on the risk of acquiring a certain disease or condition • Severity: personal perception of the seriousness of a certain disease, behaviour or condition • Benefits: personal perceptions on the effectiveness and positive consequences when adopting a new behaviour • Barriers: personal perception of the obstacles that may prevent him/her to adopt a new behaviour
Added constructs: • Cues to action: factors that trigger a behaviour • Self efficacy: personal perception on his/her ability to adopt a behaviour

HBM has been used in many health care settings in order to examine many and different health care behaviours and attitudes such as weight management [22], x-ray screening tests [44,45], sexual behaviours [46], coronary heart disease preventive behaviours [47], vaccination behaviours [48,49], diabetes management perceptions [37], nutrition [50], self breast examination perceptions [51-53], prescribed medication compliance [54], and perceptions on the Papanicolaou test [55]. It has also been previously tested and found as an appropriate theoretical model to use for measuring attitudes of nurses and health care workers towards implementing certain aspects of universal precautions from occupational exposure to pathogens [8,56,57].

### Aim

The aim of the study was to study factors that influence nurses' compliance with Standard Precaution in order to avoid occupational exposure to pathogens, by employing a qualitative research design. This paper reports factors that have emerged from this study.

## Method

### Design

The study employed a qualitative research design, with the use of focus groups. Focus groups can be used to elicit answers on a specific issue [58,59] from many individuals in a short period of time [60]. Through focused discussions, researchers attempt to study a topic of interest in depth by composing teams in which participants share a common aspect (for example, common workplace) and discuss similar experiences. These discussions intent to encourage the participants to express their feelings on the studied subject by allowing a free exchange of ideas, experiences, agreements or disagreements, in a pleasant and non threatening environment [59,61]. In addition, discussions within a group stimulate memories and exchange of ideas or opinions, leading to a more in-depth study of a subject [62,63].

### Participants

The formation of the focus groups for this study followed the guidelines of relevant literature [58,59,63]. Thirty-two nurses working in the two biggest (in terms of bed capacity) public general hospitals of Cyprus, where all medical specialities are offered, were invited to participate. Purposive sampling method was employed in order to include nurses from as many different clinical disciplines as possible. Finally, 30 nurses (r.r. 93.7%) participated (26 females, 4 males), working in general surgery, neurosurgery, plastic surgery, internal medicine, operating theatres, intensive care units, cardiology intensive care units, ear-nose-throat surgery departments, paediatric departments, paediatric intensive care units, burns units and orthopaedic departments. Of the participants 25 were female and 5 male nurses, 27 were staff nurses and 3 were senior nursing officers. In order to avoid any influence of staff nurses from the presence of senior nurses, only one senior nurse was present to three out of the four discussions (a fourth senior nurses was unable to attend to the allocated discussion). There was no information provided to the participants on the ranking of seniority among them in order to avoid any influence on junior nurses. Each group consisted of 6-10 persons (mean age 39, SD 8.6 years, mean experience in practicing nursing 17.5 years, SD 9.4 years). The criteria for inclusion were willingness to participate, two years of working experience, active provision of care to patients and hospital workplace.

The number of focus groups used was determined by the incoming of new information [59,64]. Four focus groups were contacted. The fourth focus group did not provide any new information when compared with the previous, and therefore it was decided that now more discussions were necessary, as ideas has reached saturation.

### Ethical considerations

The protocol of the study was reviewed and approved by the Cyprus National Bioethics Committee and the Ministry of Health of Cyprus. As this study is a part of a PhD thesis, a supervisory committee reviewed and approved the study's protocol. All the participants were fully informed about the purpose of the study and that the discussions would be recorded. The data that emerged were treated with confidentiality. Participation in one of the discussions was considered as informed consent.

### Process

Mean duration of the four focus groups was 84 minutes and they were conducted in a quiet location. Each participant had previously received a letter including information on the purpose of the study and the process, as well as information on the HBM. A short demographic data sheet was distributed prior to each meeting. A nurse moderated the discussions. He was fully grounded in the aims of the study. His role was to welcome, introduce, and provide an overview of the topic as well as set some ground rules. Every effort was made to provide a pleasant atmosphere, facilitate the conversation and ensure that no participant would dominate the discussions [63,65]. During discussions, the facilitator did not allow any participant to dominate the discussion, but allocated time evenly among them. All participants were encouraged to freely express their opinion. Both the facilitator and the main researcher evaluated the recorded conversations, prior to the analytical phase, and verified the above as an achieved fact. In addition, both the facilitator and the main researcher (who was present during the conversations in order to monitor non verbal responses), agreed that they did not observe any domination of any discussion by anybody. In order to ensure that the moderator could not influence by any means the discussions and at the same time to assure the validity of the study, the researchers chose an experienced moderator who was not known to the participants. This was established by using a moderator coming from an entirely different clinical area, working in a different hospital from those that the participants did. A general introductory question, which was similar for all groups, was used:

"What are the reasons that personally influence you to comply with Standard Precautions in order to avoid occupational exposure to microorganisms?"

It then progressed to more specific questions based on the theoretical framework used [59,66]. The moderator intervened whenever needed to avoid leaps and detours during the discussion. At the end of each discussion, he briefly summarized the main points of the discussion, asked if it reflected the opinion of the team, and invited further comments, corrections, or amendments. Discussions ended when the discussed subject, according to the participants' opinion, was fully covered and no more information was elicited. The principal investigator (G.E.), who was also present during the discussions, was responsible for observing and noticing all nonverbal responses of the participants (smiles face impressions, movements, head nodding, and gestures). A third person kept field notes.

### Analysis

The focus group interviews were transcribed verbatim by the main researcher. Transcripts were later supplemented with the field notes as well as the nonverbal responses that were observed during the interviews. Each final version of the transcripts was read for three times before the analysis, in order to enable the researchers to understand its content and draw an analysis plan. Analysis followed the guidelines by Krueger and Casey [59]. All members of the research team analyzed the content of the transcripts, trying to code and fit emerged themes on factors influencing compliance within the constructs of HBM.

### Quality of the study

In order to establish the quality of the study [60], the four criteria for establishing the trustworthiness of qualitative data described by Guba and Lincoln [67] were used: credibility, transferability, dependability and conformability.

**Credibility **refers to the confidence in the truth of the data produced [60]. *Prolonged involvement *was performed by the principal investigator who spent sufficient time separately with each participant prior to the formation of the focus groups in order to build trust with them, discuss the subject under study, and seek opinions, interpretations, or meanings. Information gathered was later used for preparing a sequence of questions that guided the group discussions in cooperation with the moderator of the group interviews. *Triangulation *was employed by combining group interviews, observation of nonverbal responses during these interviews, and literature review for gathering appropriate data that were later checked against each other [64]. *Participants' feedback (checks) *was sought at the end of each discussion, in order to confirm that data gathered were true to their experience.

**Transferability **refers to the extent to which the results that emerged from the sample can be transferred or generalised to the whole population [60,64,67]. In order to enhance transferability of data, a purposive sampling technique was used by inviting selected nurses from various clinical disciplines, aiming in this way to seek the opinions from participants coming from as many different working environments as possible.

**Dependability **refers to the stability of the data that emerged over time or conditions [60]. In this study, analysis of data was performed by all four members of the research team. The results of the analysis were then compared. A 90% agreement considering the distribution of the data that emerged into one of the domains of the HBM was reached. Further discussions followed in order to achieve consensus over the remaining items. As a cut-off point it was decided that a 75% agreement (absolute majority) between the researchers should have been reached in order to accept an item's distribution in one of the constructs of the HBM. If this level of agreement was not reached, then this item was discarded. Based on this criterion, the researchers did not agree on 3 items that emerged from the discussions, therefore these were discarded.

**Conformability **refers to the characteristics of the data, mainly their objectivity and neutrality [60]. The researchers addressed this issue by developing an audit trail [60] in order to achieve conformability. This audit was separately performed by all four members of the research team so as to enhance its quality. Appropriate measures were taken when necessary.

## Results

Analysis of data revealed many factors that may influence nurses' compliance with Standard Precautions in order to avoid occupational exposure to microorganisms

### Barriers

#### Emergency situation

Many participants described an emergency situation as a major obstacle in following precautions: A male nurse working at an intensive care unit said: "... the emergency, something unexpected may happen, an emergency situation may occur [...] you do not have the time to use protective equipment." The participants argued that when nurses come across situations of life or death, they will ration their time to provide care instead of taking time to using protective equipment, despite the fact that this may expose them to microorganisms. Their main concern is to protect the patient's life. One of the female nurses working at a cardiology intensive care unit explained: "... if it is a matter of life or death, you see the patients having a serious bradycardia or hypoxia, you only think how to save him/her." And another female nurse working at a burns unit said: "... yes, if I judge that the patient needs help to stay alive, it will not be my main concern to use gloves..." And yet another one said: "... we had to rescue the patient, we neglected our own safety."

#### Availability of equipment

Another factor perceived as a barrier was the lack of protective equipment available (masks, gloves). The participants stated that they often come across situations where they must use protective equipment, but this is not possible due to the lack of availability of such equipment. A nurse working at an orthopaedic ward said: "Many times we want to use protection, but we cannot because [protective equipment] is not available [...] and this is a common phenomenon." Another female nurse argued: "... we were eighteen nurses at the intensive care unit on a morning shift, and there was not a single pair of gloves available to provide nursing care. And this happened not because the senior nursing officer failed to order supplies. No ... this happened due to the fact that no gloves were available to be ordered."

A different aspect of non availability of equipment is the storage of such equipment in places far from where nursing care is provided. A nurse working at a plastic surgery department said "... you must have the equipment at your disposal immediately, at the time you need it. Usually, it is stored in places not close to the patients' rooms. In this case, I may provide care without protection rather than to try to find it in a warehouse."

Another parameter is the fact that this equipment may be available, but not in sizes or types that are necessary. A female nurse, working at a burns unit argued: "... I wear size "small" of gloves. Usually, this size is not available, because not many health care professionals use them, and therefore they are not usually ordered. I try to use other sizes, but I cannot work if I use "medium" or "large" sizes. Therefore, I prefer not to use them at all."

#### Negative influence of protective equipment on nurses

The groups expressed the idea that the use of protective equipment reduces nurses' skills (for example, to perform venipuncture when wearing gloves). Similar statements as the one that follows were reported by many participants: "... using gloves to draw blood from a patient reduces my dexterity, I cannot feel the vein because the gloves interfere" (female nurse working in an internal medicine department). It was anticipated that by using protective equipment, nurses' work performance is impeded and that they cannot perform certain procedures. Even though these procedures are known to possibly expose them to microorganisms, the reduction of skills that occurs with the use of protective equipments, negatively affects the compliance with Standard Precautions. One of the male participants stated: "I cannot do my job when wearing gloves. I see colleagues wearing gloves even to make up beds. I cannot, I do not like gloves [...] I cannot palpate a vein [...] they reduce my skills, I cannot work." In addition, a female participant working at an intensive care unit responded that the use of personal protective equipment makes them feel uncomfortable: "... I cannot breathe normally when I wear a face mask [...], it has an awful smell. I prefer not to wear a mask, even when its use is necessary." Furthermore, many respondents said that the use of protective equipment negatively influences nurses' health status: "... the use of gloves irritates my hands."

#### Patients' discomfort

Patients' discomfort was considered as a major obstacle to following Standard Precautions. The participants pointed out that patients may experience distress, anxiety, or even sorrow when a nurse offers nursing care while covering himself/herself with a mask or gown or while using gloves. In addition, they may anticipate these measures taken by nurses as an indication that their health care status is not good or is getting worse: "... the negative impact on the patient's psychology may have the use by me of a face mask every time I go to offer a bed bath" (respond from a female nurse working at an orthopaedic department). The groups suggested that the way nursing care is provided can sometimes offend the patient, if the means of protection is used very often, although this may be necessary under the requirements of Standard Precautions.

#### Too busy, lack of nursing personnel, implementation of guidelines is time consuming

The groups identified three similar factors that are perceived as obstacles to following Standard Precautions. Often nurses come across many responsibilities to be fulfilled. This leads nurses to avoid the use of Standard Precautions, even when it is anticipated that they may be exposed to microorganisms. A nurse working at the cardiology intensive care unit said: "... I am very busy, I have to do this and that and there is not enough time. So I will choose to avoid doing certain things, and one of them is to reduce the prevention measures meant for my safety. There is not enough time to put on gloves...." Another participant, working at the burns unit, agreed and added: "I agree, we are often too busy to take precautions. But why are we busy? In my opinion, because there are not enough nurses to perform nursing duties...." In addition, many participants argued that following Standard Precautions in many cases is time-consuming (for example putting on a gown).

#### Provision of nursing care to children

An interesting factor that emerged from the groups-not described in relevant literature so far-is the provision of nursing care to children. This age group was anticipated as low risk, and therefore, the use of a preventive measure was considered as unnecessary. This opinion was mainly offered by nurses working in paediatric departments: "... to treat a child, to help a child move from bed to a chair [...], it is exaggeration to put on gloves or masks...." A female participant argued that: "... children are so innocent, it is unlikely that they would suffer from a contagious disease [...], a child can vomit in my hands and this may not worry me." Even when children are hospitalized suffering from contagious diseases, participants agreed that they, in many cases, do not use protective equipment when treating them in order to avoid making the child or his/her relatives feel bad: "... in one case I wore gloves to take blood from a child. The mother gave me an angry look, asking me to remove gloves because her child was not suffering from any disease." Surprisingly, most participants admitted that they were aware of the fact that Standard Precautions require that all patients, including children, should be treated as contagious and that children can be carriers of serious infectious diseases. Nevertheless nursing children was considered as a major barrier to follow guidelines.

#### Influence on nurses' appearance

A female participant suggested that the use of protective equipment has a negative impact on her appearance. Many members of the groups (not only women) agreed with this idea, saying that they would prefer not to take precautions if the use of appropriate equipment would have a negative impact on their appearance: "... my appearance is very important to me. If I wear a hair cap, this will destroy the look of my hair. I spend a lot of time making my hair look the way I want them to look, and I am not going to let anything damage this." Another female participant argued: "... the use of face mask damages my lipstick and makeup. I prefer not to use it [the face mask]."

#### Psychological factors

Many participants mentioned several psychological factors that may affect a nurse's decision to follow standard precautions. Nurses may feel embarrassed to follow guidelines, especially if these are not routinely used in the department they work in. In addition, the negative behaviour, regarding complying with the use of personal protective equipment, displayed by more experienced colleagues may affect younger nurses' compliance. A junior female staff nurse argued: "... if I see that my supervisor does something, for example take blood from a patient without using gloves, I will probably be influenced as well by this practice" and "... relatives sometimes look at me with a strange look when I put on a gown or a face mask. This makes me feel unhappy [...] and embarrassed because they think I am overly fearful." Another male participant said that he feels strong and healthy, and therefore it is unlikely that he would acquire a disease during his contact with patients: "... I exercise, take vitamins [...] I am in perfect condition. I feel that I am well protected."

#### Working experience as a nurse

It was anticipated by the participants that when nurses gain enough experience, they are very confident about their capabilities. Therefore, certain guidelines may not be followed, as argued by a nurse with considerable clinical experience: "...the more capable I feel, the less preventive measures I may take."

##### Physician's *influence*

Interestingly, some participants said that they may be influenced by how physicians work: "... [physicians] do not wear gloves when they draw blood or examine a patient." Nurses may even follow the demand of a doctor: "The doctor forbids the use of gloves and masks. I am afraid to say I disagree" (response from a female junior nurse working at a neurosurgery department).

### Susceptibility

#### Risk of being infected

The groups argued that nurses are continuously exposed to microorganisms. The participants explained that several nursing procedures (for example, giving bed bath or starting an intravenous line) require contact with patients. One female participant said: "... we work in an environment full of microorganisms. It is easy to get infected [...] I consider my personal safety as very important". Nurses acknowledge the fact that they work in an unhealthy environment and provide care to people that may suffer from contagious diseases. Additionally, the participants argued that they are worried about the fact that they may transmit a health care acquired infection to a member of their families. A married nurse said "... my family is in my heart. I do not want them to suffer from a disease that I may bring home from my workplace" and "... I will assure my personal safety first [to avoid being infected] in order to be sure that my family will be safe as well." This was acknowledged by many other participants.

#### Vulnerable to diseases

Some participants explained that they need to take preventive measures because their immune system is not strong enough; therefore, it is very easy for them to become infected due to the contact with sick people: "... I am vulnerable [...], I will use protection in any case. I do not want to become sick. Believe me, I get sick very easily and I mean really sick." Nevertheless, the fact that some nurses feel vulnerable may lead to extraordinary measures: "... I often use more than what is required, I know that this is not necessary, but I am afraid" (female nurse working in a general surgery ward).

### Benefits

#### Protection from being infected

The participants agreed that by implementing the requirements of Standard Precautions in their daily practice they are protected. A nurse working in an internal medicine ward said "... they can protect me [the Standard Precautions] ... I have read a lot about them [protective equipment] and I am confident that I am well protected." The term protection was not only limited to their own protection but also to their families' as well.

#### Psychological factors

The groups argued that they worry a lot about the fact of being due to exposure to microorganisms. It was explained that a good reason to follow standard precautions is the fact that by implementing them, their anxiety about becoming infected is dramatically decreased, because protective equipment can serve as a barrier to the transmission of microorganisms. Therefore: "... I will be calm, both at work and home, knowing that I do not need to worry about being infected [...] because I follow the instructions", one female nurse, working at an orthopaedic department argued.

### Cues to action

#### Provision of nursing care to adult patients

Provision of care to adults was considered by the participants as a major factor that positively influences them to use Standard Precautions. Adult patients were described as a "high risk group". When the participants were asked to explain the difference in perception between children and adult patients, it was difficult for them to give a reasonable explanation other than the fact that children are "... innocent creatures, well protected by their parents and it is unlikely that they have been exposed to a disease" (nurse working in a paediatric department) whereas adults "... are independent persons, there is much more chance for them to be exposed to and carry an infectious disease" (nurse working in an adults intensive care unit). One participant, who worked in an ear-nose-throat department, where children and adults are nursed, said: "... it is easy to forget or not think of protection when you have a child in your hands. But it is different when you have an adult."

#### Previous exposure

Being exposed to a microorganism was agreed to be a devastating experience. The exposed person needs to follow certain examinations, and if necessary pharmaceutical regimes. The psychological impact can be high (anxiety, depression) both for the nurse and his/her family: "... I had this patient at the intensive care unit. I used to draw blood from arterial lines without gloves. I believed that I was experienced and this would protect me from being exposed. But sometimes, yes, I was exposed by this and other patient's blood. And then, we learned that this patient was HIV positive. I was shocked, panicked [...], I thought now what? [...] a thousand thoughts, for myself, my family, have I exposed them as well?" (argument by a nurse working in an adults intensive care unit).

#### Continuous reminding-continuous education-guidelines

The participants believed that continuous reminders about the need for implementing Standard Precautions, improve compliance. Distributing leaflets among nursing personnel, scattering small posters in various places of wards, and continuous reminders from senior nursing officers about the benefits of complying with Standard Precautions and the possible consequences of the exposure to microorganisms was considered to be a useful way to keep nurses in line with protective guidelines. In addition, the participants emphasized the need for continuous education. They argued that new instructions and new methods and equipment for protection should be immediately available to them by means of educational programs: "...the lack of information, how and when to take appropriate protective measures, influences me. If I do not know how to use something new or when to use it, how can someone expect me to make use of this it?" Furthermore, the participants pointed out the need for developing guidelines that would help them decide when and which protective equipment was appropriate.

#### Patients' personal characteristics

Patients' appearance was believed to be a serious factor that may lead a nurse to comply with standard precautions. More specifically, it was argued that if a patient is dressed carelessly, has tattoo in many places of his/her body, has low personal hygiene status or low educational level, then he/she would be considered as a high risk for carrying an infectious disease: "If I see a person full of tattoos, I will say this is not normal, probably this patient lives an extraordinary life. You know what I mean? So I will take extra caution with him/her." And another female participant said: "I am influenced by the way a patient looks. If he/she is clean, I will say OK the chances for him/her being a carrier of a disease are limited, but if he is dirty and not well dressed then things are different."

#### Provision of nursing care to foreign patients

The participants offered the provision of nursing care to foreign patients coming from less developed countries as a factor that persuades them to follow Standard Precautions. It was argued that patients coming from countries with different (often insufficient health care systems) may more often suffer from infectious diseases more often. For this reason, more preventive measures are likely to be taken when nursing this group of patients.

#### Significant others

The groups argued that when Standard Precautions are followed by colleagues with more knowledge or by senior nursing personnel, then they are influenced to comply as well. A junior nurse said "My supervisor uses gloves when she starts an intravenous line; I will certainly follow her example." In addition, it was pointed out by the participants that when the senior nursing officer "demands" the application of Standard Precautions - as means of "pressure" over them - they are "influenced" (obliged) to use them.

### Severity

#### Fear

One female participant said that she gets terrified every time she comes across the idea that she may get infected by a disease when practicing her nursing duties. This fear becomes worse when she thinks about her family: "I will never forgive myself if one of my children suffers from a disease for which I am responsible for transmitting [...] I am terrified even thinking about this [...] to avoid this, I always take precautions."

#### Serious disease-death-negative impact on life

Many hospital acquired infections may be serious, even fatal (for example, AIDS). The participants said that because they think of the possibility of dying due to a disease acquired from a patient, they are influenced to take appropriate preventive measures.

#### Costs from being infected

Many participants argued that becoming infected from a serious hospital-acquired infection may jeopardize a lot of important things in their personal life: "... my career may end, my self-esteem will be seriously affected [...] and "I will not be able to look my family in the eyes any more if a member of the family suffers from a disease that I brought home" (male nurse).

### Self-efficacy

Many participants said that it is difficult for them to change their behaviour, even though they know that it is not correct. This was mainly argued by older nurses: "We have been trained to work as we do now. For example, we were trained not to use gloves when giving bed bath or making wound changes. It is difficult after so many years to change" and "I do not think that after so many years of practicing the way I do, I will be able to change [...] I cannot."

## Discussion

To our knowledge, this is the first research study contacted in Cyprus among any group of health care workers, investigating the issue of compliance with Standard Precautions to avoid occupational exposure to pathogens. The HBM has been previously used as a theoretical framework in many studies, and has been successful in explaining a variety of human behaviors and attitudes, including compliance with Universal Precautions, the previous version of Standard Precautions. Therefore the use of the HBM as a sound and useful theory, improves the internal validity of this study, and offers the ability for comparison among similar studies.

This study examined the factors that influence nurses' compliance with Standard Precautions in order to avoid occupational exposure to microorganisms. Using the HBM as theoretical framework, this study has concentrated on those factors that affect compliance either negatively (barriers), leading to non compliance, or positively, leading to compliance.

Many of the emerged factors, contributing to non-compliance, are in accordance with findings of previous studies. Nevertheless, most of these studies have focused only to those factors that negatively influence compliance and lead to non compliance or have not used a theoretical model as background [7,10,23,29,30,41]. In addition, most of them used questionnaires for gathering their data; therefore the results must be cautiously compared with the findings of this study which employed a qualitative design.

Non-availability of equipment was reported as an obstacle for implementing Standard Precautions, as they cannot be followed if the health care worker does not have direct access to them. In fact, some participants argued that equipment is stored or even locked far away from the place nursing care is provided, making their use impossible under certain situations (for example emergency situation). Similar finding was reported in other studies [8,31,68-70]. It is therefore vital for nurses to have the protective equipment at their disposal, for use when necessary.

Previous studies showed that negative influence on nurses, for example skin irritation [31] or hand pain from the use of gloves [71] were also factors inhibiting health care professionals from implementing precautions. Similar findings have been reported by the participants of this study. This negative influence can, to a certain extent, be overcome, by using for example high quality products (for example soap). This is a matter of policy, and health care policy makers should take this into consideration, if they want to avoid unnecessary sick-leaves or low level of nursing care.

The participants in the focus groups argued that many times there are time restraints to implement precautions. They reported that due to heavy workload, there is no time to follow guidelines, even if they want to. In addition, dealing with an emergency situation was also considered as a factor inhibiting nurses from implementing Standard Precautions. It was explained that an emergency situation requires doing a lot of things at the same time, very fast and usually under heavy pressure. This situation - as it was argued - may influence nurses not to follow the protective guidelines due to insufficient time (several conditions may be matters of life or death). Lack of time has also been widely described elsewhere [7,29,31,68,72] as factor not facilitating the implantation of precautions.

Participants reported that certain equipment interferes with skills (for example use of gloves decreases dexterity when drawing blood), therefore they prefer not to follow them. Although this behaviour may help them provide care more easily or efficiently, nurses should have in mind that is leaves them without protection against pathogens; therefore the use of protective equipment should always be implemented. This report supports previous findings in the literature [8,23,29,68,70,71,73] where also negative influence on skills was reported as a barrier for following precautions.

This study has also revealed many factors that negatively influence nurses' compliance rarely been described before in relevant literature. Provision of care to children was perceived as a barrier to implement Standard Precautions. It was assumed that children are low-risk patients, although it was acknowledged that they can also carry contagious diseases. Physicians were described by participants as non compliers with precautions; nevertheless, nurses admitted that they are influenced by how physicians work (without precautions) or even follow their demands for not implementing precautions. An interesting factor that the participants believed contributed as a barrier was the negative impact of protective equipment on the nurses' appearance. Female nurses argued that they would prefer not to use a face mask because it would ruin their make-up and lipstick or to use a hair cap because it would damage their hair appearance. Changing of current behaviour was also considered as a major obstacle in following the guidelines. The participants admitted that they were not willing to or capable (self-efficacy) of altering their current practice because that was the way they were trained or used to.

Many factors can positively contribute to the implementation of Standard Precautions, and are in accordance with previous findings [8,56,57]; it should be stated however, that studies focussing on factors positively influencing compliance are limited. Such factors correspond to the Benefits, Cues to Action, Susceptibility and Severity constructs of the HBM. They lead or «force» nurses to follow Standards Precautions: nurses may be obliged to do so (supervisor's instructions), or because they fear that they or their family might be at risk of being infected if do not take necessary precautions. Continuous reminders and continuous education on precautionary measures and when they should be used was also considered as an important factor for improving compliance. Interestingly, some patients' personal characteristics (low personal hygiene status, body tattoos, foreigners) were also reported as being factors persuasive for complying, despite admittance that Standard Precautions should be implemented for every patient.

### High/low influential factors for implementing precautions

By further analysing data, the authors created two influential hierarchy scales, one by type of patients and one by activity/situation (Table 2). The developed scales rank the emerged factors, starting from the less influential factor for influencing a nurse to comply with precautions, and finishing at the most influential factor. By examining these scales, clear conclusions can be made for which factors nurses consider (perceive) as more important for adopting Standard Precautions. As far as the type of patient is considered, nurses seem to believe that children are the lowest risk group; therefore they place them at the top of the relevant scale. Adult patients can be found at the bottom of this scale, making them as the highest risk group of patients. It can be concluded, based on this hierarchy, that nurses are more likely to adopt Standard Precaution when treating any adult patient. The activity that the less influence on nurses to adopt Standard Precautions is the physician's way of working/demands; therefore, this factor is located at the top of the activity/situation scale. At the bottom of this scale is death; the fear of dying due to an occupational exposure to a pathogen, influences nurses to comply with Standard Precautions. Based on their perception, death is the most influential factor that leads to the adoption of precautions. Further statistical analyses, using larger samples are needed to verify these conclusions. It should be stated however that such a hierarchy by no means suggests that those factors that appear at the top of each scale are not important. On the contrary, Standard Precautions clearly mandate that they should be implemented for all patients and under all circumstances [4].

**Table 2:** Ranking of less/more influential factors

Influential hierarchy by type of patient^a^ 1. children 2. foreigners (irrespective of age) 3. patient's personal characteristics (irrespective of age) 4. adults
Influential hierarchy by activity/situation^b^ 1. physicians' way of working/demands 2. wrong routine practice at workplace 3. patient discomfort 5. embarrassment 6. reminding for using precautions 7. lack of time 9. time consuming 10. negative impact on nurse 11. equipment not immediately available 12. non-emergency situation 13. colleagues with more experience 14. previous exposure 15. protection offered by precautions 16. cost from being infected 17. fear 18. death

a. 1 (=less influential) → 4 (=more influential)

b. 1 (=less influential) → 18 (=more influential)

### Focus groups

Focus groups are widely considered as a means to reveal feelings, ideas, perceptions, viewpoints, exchange of ideas, and thinking on a specific issue [59,60] via social interaction [64]. It was the intention of this study to seek the contribution of nurses on the issue, in order to gather the appropriate information directly from the target group (nurses), through discussions, asking them to express what they really believed and felt on this issue. Nurses from different clinical disciplines had the chance to discuss, exchange ideas, debate, argue, agree or disagree. Through this process, numerous new ideas were revealed. Some of the factors believed to influence compliance or non compliance with Standard Precautions, in order to avoid occupational exposure to microorganisms have never been reported before. The barriers construct of the HBM incorporated most of the factors, which represent those that may not allow nurses to conform to the guidelines.

## Limitations

Since this is a qualitative study, its results cannot be generalised to the population of nurses [64,74]. Further studies with larger samples and mix methodologies should be conducted. In addition, there was a geographical location limitation. The participants in this study worked in only two selected hospitals, based on the fact that these hospitals were the biggest in Cyprus, incorporating all medical specialities, as well as because of their proximity to the premises that the discussion took place (in order to enhance the participation) Every effort was made to include participants working in various nursing disciplines.

The facilitator, following the instructions of the research team, did not offer any ideas for discussion, but alternatively, asked for deeper discussion of the issues that were described by the participants. As a result, this paper reports only the findings as they emerged during the discussions as offered by the participants. By doing this, some factors that may influence nurses' compliance with Standard Precautions may have not been reported (for example using Standard Precautions to prevent the transmission of microorganisms to colleagues).

## Conclusions

In general, the participants acknowledged the value of Standard Precautions as a means for providing protection against occupational exposure to microorganisms. They also accepted that several factors may contribute to their decision to comply or not with Standard Precautions; some of the described factors may be out of the nurses' control. Adopted behaviour may be influenced by the balance of these positively or negatively leading factors. If those factors that lead to noncompliance overcome those that lead to compliance, then it is unlikely that Standard Precautions would be followed. Therefore, it is necessary to reveal those factors that influence compliance (positively and negatively), and develop plans in order to eliminate those that do not allow the implementation of Standard Precautions and promote those that do.

The data that have emerged from this study, as well as information coming from published literature and discussions with experts on hospital acquired infections, will be used for the development of a questionnaire, based on the HBM, examining the factors that influence nurses' compliance with Standard Precautions in order to avoid occupational exposure to micro-organisms. It is anticipated that this questionnaire - when distributed to larger samples - will offer the opportunity to improve the compliance of nurses with Standard Precautions, in order to minimise occupational exposure to microorganisms and improve nursing as well as patients' outcomes related to infection control. The results can be used by nurses, hospital managers, policymakers, risk managers, and nurse educators as a means of health promotion among nursing personnel.

## Competing interests

The authors declare that they have no competing interests.

## Authors' contributions

All authors read and approved the final manuscript. GE was responsible for the study conception and design, formation of the focus groups, data synthesis and analysis and manuscript drafting, EP was responsible for the study conception and design, data analysis, critical revisions of the paper and study supervision, VR was responsible for the study conception (use of HBM), data analysis, critical revisions of the paper and study supervision, and AM was responsible for data analysis, critical revisions of the paper and study supervision.

## Pre-publication history

The pre-publication history for this paper can be accessed here:

http://www.biomedcentral.com/1472-6955/10/1/prepub
